# Differential engagement of brain regions within a ‘core’ network during scene construction

**DOI:** 10.1016/j.neuropsychologia.2010.01.022

**Published:** 2010-04

**Authors:** Jennifer J. Summerfield, Demis Hassabis, Eleanor A. Maguire

**Affiliations:** Wellcome Trust Centre for Neuroimaging, Institute of Neurology, University College London, 12 Queen Square, London WC1N 3BG, UK

**Keywords:** Scene construction, fMRI, Episodic, Memory, Future thinking, Hippocampus, Autobiographical, Objects

## Abstract

Reliving past events and imagining potential future events engages a well-established “core” network of brain areas. How the brain constructs, or reconstructs, these experiences or scenes has been debated extensively in the literature, but remains poorly understood. Here we designed a novel task to investigate this (re)constructive process by directly exploring how naturalistic scenes are built up from their individual elements. We “slowed-down” the construction process through the use of auditorily presented phrases describing single scene elements in a serial manner. Participants were required to integrate these elements (ranging from three to six in number) together in their imagination to form a naturalistic scene. We identified three distinct sub-networks of brain areas, each with different fMRI BOLD response profiles, favouring specific points in the scene construction process. Areas including the hippocampus and retrosplenial cortex had a biphasic profile, activating when a single scene element was imagined and when 3 elements were combined together; regions including the intra-parietal sulcus and angular gyrus steadily increased activity from 1 to 3 elements; while activity in areas such as lateral prefrontal cortex was observed from the second element onwards. Activity in these sub-networks did not increase further when integrating more than three elements. Participants confirmed that three elements were sufficient to construct a coherent and vivid scene, and once this was achieved, the addition of further elements only involved maintenance or small changes to that established scene. This task offers a potentially useful tool for breaking down scene construction, a process that may be key to a range of cognitive functions such as episodic memory, future thinking and navigation.

## 1 Introduction

Recalling past experiences, imagining fictitious and future events, and navigating in large-scale space share a set of brain regions in common that has come to be known as a ‘core’ network (Addis, Wong, & Schacter, 2007; Hassabis, Kumaran, & Maguire, 2007; Spiers & Maguire, 2006; Szpunar, Watson, & McDermott, 2007). This network overlaps considerably with the default or resting state network (Buckner, Andrews-Hanna, & Schacter, 2008; Raichle et al., 2001) and comprises the hippocampus, parahippocampal gyrus, retrosplenial cortex, posterior cingulate cortex, precuneus, temporo-parietal junction, angular gyrus, lateral temporal cortex, ventrolateral prefrontal cortex, and medial prefrontal cortex (Spreng, Mar, & Kim, 2009). Several theories have been advanced to explain the functioning of this core network (Buckner & Carroll, 2007; Hassabis & Maguire, 2007; Schacter & Addis, 2007). We have put forward one such proposal, suggesting that the core network is concerned with ‘scene construction’, the process of mentally generating and maintaining a complex and coherent scene or event (Hassabis & Maguire, 2007; Hassabis & Maguire, 2009). This is achieved by the reactivation, retrieval and integration of relevant semantic, contextual and sensory components, stored in their modality-specific cortical areas, the product of which has a coherent spatial context (Hassabis, Kumaran, Vann, & Maguire, 2007), and can then later be manipulated and visualised. Scene construction differs markedly from simple visual imagery such as that for single objects, in that it requires the flexible association and integration of many scene elements. We proposed that scene construction is common to a range of disparate cognitive functions including episodic memory, imagination, episodic future thinking, and navigation (Hassabis & Maguire, 2007; Hassabis & Maguire, 2009).

Focusing on the core network and scene construction throws up some fundamental questions, such that even if one does not subscribe to the idea of scene construction, are nevertheless important to consider. For instance, what are the components or elements that come together to allow us to recall the past or imagine the future? One key component is the individual objects that make up the imagined scenes and events. Having established that a core network was active when participants imagined fictitious experiences, in the same study Hassabis, Kumaran, and Maguire (2007) had participants imagine single acontextual objects. Thus, the objects were devoid of background or context, rather than imagined as part of a scene. The associated brain activations comprised the lateral occipital complex (LOC), intra-parietal sulcus (IPS) and dorsolateral prefrontal cortex (DLPFC) (Summerfield, Hassabis, & Maguire, 2009 also found this result for their object conditions – personal communication; see also Sugiura, Shah, Zilles, & Fink, 2005). The differences between this simple object network and that for complex scene construction, suggest they represent dissociable cognitive processes with distinct neural bases. But clearly, complex scenes and experiences are constructed out of simpler elements, so a key question remains, how is the transition made between this object network and the core scene construction network?

It is notable that in previous neuroimaging studies examining the imagination of fictitious or future experiences, participants were given a general cue [e.g. *5 years in the future; dress* (Addis et al., 2007); *imagine lying on a white sandy beach* (Hassabis, Kumaran, & Maguire, 2007)]. This was followed by a period of unconstrained imagining over a timescale of between ∼10 and 60 s (Addis et al., 2007; Botzung, Denkova, & Manning, 2008; Hassabis, Kumaran, & Maguire, 2007; Okuda et al., 2003; Szpunar et al., 2007), resulting in activation of the core network. In order to examine how scenes are constructed out of component parts, we devised a novel task. Instead of having participants imagine a full scene or scenario right off the bat, they had to construct it element by element, thus slowing the process down. Moreover, because the elements were provided by the experimenters, this lessened the likelihood of reliance on specific personal episodic memories. In addition, we manipulated the number of elements supplied on a given trial, allowing us to also examine the effect of increasing scene complexity.

The aim of study was not to try and ascertain the roles of specific brain regions in scene construction. Rather, our goal was to understand more about how the transition is made between the single object (element) network and the core (scene construction) network, to perhaps break these networks down into smaller sub-networks and examine how they might be engaged at different points in the scene construction process, thus providing additional clues about how episodic memories and simulations of the future are (re)constructed by the brain (Bartlett, 1932; Conway & Pleydell-Pearce, 2000; Hassabis & Maguire, 2007; Rubin, Schrauf, & Greenberg, 2003; Schacter, Addis, & Buckner, 2008; Schacter, Norman, & Koutstaal, 1998).

## 2 Methods

### 2.1 Participants

Nineteen healthy right-handed native English speakers took part in the study (8 females; mean age 25.7 years, *SD* 4.7). All participants had normal or corrected to normal vision and gave informed written consent in accordance with the local research ethics committee.

### 2.2 Task and procedures

The fMRI task comprised construction trials and matched control trials. In construction trials participants gradually built up simple naturalistic scenes in their imagination based on a series of auditorily presented phrases. In control trials, participants attended to a series of auditorily presented phrases designed to elicit minimal imagery and mnemonic associations.

#### 2.2.1 Construction trials

In construction trials, participants listened to a series of short spoken phrases, one after the other. Phrase duration varied between 1 and 3 s (this was balanced across all element positions), and thus allowed for jitter in the experimental design. Participants had to imagine the object or background feature (“element”) that each phrase described, with their eyes closed. Each phrase described one element. Objects were everyday items found within an indoor environment. Backgrounds were static features integral to an internal environment (e.g. wall, floor). Each object or background element was accompanied by one or two adjective descriptors (e.g. colours, patterns, material) to ensure that each element was distinct.

There were four construction conditions in which the number of phrases, and thus the number of elements, was manipulated: 3, 4, 5 or 6 elements (see Table 1 for example stimuli). For any given trial, participants were instructed to integrate the elements together in a realistic manner so as to construct a spatially coherent naturalistic indoor scene in their imagination. Elements were grouped together within a trial to be qualitatively complementary in nature (e.g. matching colours, patterns) to aid vivid imagination but were not specifically contextually related. The order of objects and backgrounds within each trial was counterbalanced to minimize order effects. In addition, the allocation of objects which typically might be located on another object (often smaller objects, e.g. a book – on a table), and objects which might typically be found on a background (often larger objects, e.g. a chair – on the floor) was also counterbalanced across trials, as was the type of background feature (e.g. wall, floor, ceiling).

During the task, participants adhered to several important instructions and this was verified in the post-scan debriefing session. They were instructed to start with a ‘clean slate’ and imagine the first element as acontextual, i.e. they had to visualise the element either against a plain background or simply floating in mid-air, in the absence of any background context. They were told to imagine only what the narrative described and not to include any additional objects or associated background contexts. Participants were required to construct the scene in a first-person perspective, in the centre of their imagined field of view, but with themselves absent from the scene. They were instructed not to change their viewpoint or perspective as the construction unfolded in their imagination. Finally, participants were told not to retrieve familiar objects/backgrounds, and also to create novel scenes, distinct from familiar internal environments (e.g. their own house).

#### 2.2.2 Control trials

In control trials, participants heard a series of short “jargon” phrases, one after the other, with their eyes closed. These controlled for basic auditory stimulation, language, attention, working memory, difficulty, and effort (see Table 1 for example stimuli). Phrases were qualitatively similar in nature and were made up of word combinations designed to elicit minimal imagery and mnemonic associations. To ensure that participants paid attention during these trials, they were informed that at the end of each trial they would be asked whether a particular word had been spoken in one of the phrases in that trial. Importantly, during control trials, participants were instructed not to visualise any related images, make any judgements or form opinions, or recall any memories associated with the phrase content. In addition they were instructed not to use any specific strategies to remember the words. There were four control conditions in which the number of phrases in a trial was either 3, 4, 5 or 6. These were matched in duration and delivery to the imagination trials (phrase duration ranged from 1 to 3 s).

#### 2.2.3 Trial design

**Fig. 1:**
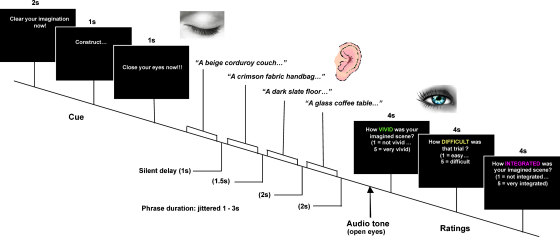
Task design. Timeline of an example trial, in this case a construction trial with four elements. See Section 2 for full details.

Fig. 1 shows the timeline of a typical trial. Each trial began with the visual presentation of the text “Clear your imagination now” (2 s). Participants were instructed to begin each trial with a clear imagination and simply imagine a “blank slate” in readiness for the onset of the phrases. Following this, the cue “Construct…” (for construction trials) or “Attend…” (for control trials) was presented (1 s). This was then joined by the text “Close your eyes now!” (1 s), requiring the participant to close their eyes. Participants then heard the series of 3, 4, 5 or 6 elements. They did not know in advance how many elements they would hear on any given trial. There was a short silent delay between each of the elements (1 s after element 1; 1.5 s after element 2; 2 s after element 3; 2 s after element 4; 2 s after element 5; and 2.5 s after element 6) to ensure vivid imagining and integration of the objects and backgrounds (these times were selected to be the most appropriate after extensive pilot testing). After the narrative had finished, an audio tone (1 s duration) signalled the participant to open their eyes. Following this, in construction trials, participants performed three different ratings using an MRI compatible 5-button keypad: how difficult they found the trial (1 = easy…5 = difficult), the vividness of their imagined scene (1 = not vivid…5 = very vivid), and the perceived degree of integration between the elements (1 = not integrated…5 = very integrated). In control trials, participants were asked whether a particular word was present or absent in the trial “Was the word X in that trial?” (1 = yes…2 = no). In 50% of trials the word had been present in the trial. For “Yes” trials, the phrase position from which the probe word was taken was counterbalanced across trials. Following this, participants then rated trial difficulty (1 = easy…5 = difficult). Participants had 4 s to make each response.

There were 80 trials in total in the experiment: 40 construction trials (10 trials per condition) and 40 matched control trials. Trial types were randomly intermixed through each run and across the whole experiment. Participants first performed a behavioural practice outside the scanner with stimuli not used in the main experiment.

#### 2.2.4 Post-scan debriefing

Immediately after the scanning session participants were thoroughly debriefed. The debriefing was digitally recorded for later verification purposes. The experimenter discussed general task performance with each participant and then focussed in more detail on each trial type in turn. For construction trials, participants performed ratings (range 1–5 – see Section 3), including the detail in the image, the ease of spatial integration, the degree of maintenance, the feeling of scene coherence, how much they kept to the narrative or added additional details, the novelty or familiarity of the scene, and the involvement of the self. For control trials, participants rated imageability of the phrases, and the degree of memory elicitation.

### 2.3 Scanning parameters

T2*-weighted echo planar images (EPI) with blood oxygen level-dependent (BOLD) contrast were acquired on a 1.5 tesla Siemens AG (Erlangen, Germany) Sonata MRI scanner. Scanning parameters were selected to achieve whole brain coverage: 45 oblique axial slices angled at 30 degrees in the anterior–posterior axis, 2 mm thickness (1 mm gap), repetition time 4.05 s, TR 90 ms, TE 50 ms, field of view 192 mm, 64 × 64 matrix, in-plane resolution 3 × 3 mm. The first six ‘dummy’ volumes from each session were discarded to allow for T1 equilibration effects. A T1-weighted structural MRI scan was acquired for each participant after the functional scanning sessions.

### 2.4 Data analysis

Data were analysed using the statistical parametric mapping software SPM5 (www.fil.ion.ucl.ac.uk/spm). Spatial preprocessing consisted of realignment and normalization to a standard EPI template in Montreal Neurological Institute (MNI) space with a resampled voxel size of 3 mm × 3 mm × 3 mm, and smoothing using a Gaussian kernel with full width at half maximum of 8 mm. After preprocessing, statistical analysis was performed using the general linear model. The experiment had 4 construction conditions: (3, 4, 5 and 6 elements). These were compared against the 4 matched control conditions (3, 4, 5 and 6 phrases). Each element (or phrase) of each condition was modeled separately (36 regressors of interest in total). The time period of interest was that during which a phrase was spoken and included the short silent “visualization” period immediately following each phrase (1–2.5 s). These periods were modeled as a boxcar function and convolved with the canonical haemodynamic response function to create regressors of interest. Not only did the inter-element intervals vary between 1 and 2.5 s, the stimulus onset asynchrony was jittered because the audio phrases that described the elements varied in length from 1 to 3 s. For example, the difference in time between onset of element 1 and onset of element 3 was jittered between 5.5 and 9.5 s. It is also important to note that we did not make inferences about the amplitude of response to individual elements, rather our effects related to the change in amplitude from one element to another at a particular point. Because our analyses were predicated upon contrasts between elements, significant results were only possible if there was a unique amount of experimental variance in the response.

Participant-specific movement parameters were included in the design as regressors of no interest. Participant-specific parameter estimates relating to each regressor were calculated for each voxel. These parameter estimates were entered into a second level random-effects analysis using standard repeated-measures analysis of variance (ANOVA) analyses. Average behavioural rating scores for difficulty, vividness, and integration were calculated for each condition and included as contrast weights and analysed using standard *t*-test analyses. *P* < 0.001 uncorrected, with a minimum cluster size of 5 voxels, was considered the criterion for significance in areas of *a priori* interest (see brain areas comprising the object and core networks described in the Introduction), but we report all activations at *p* < 0.001 for completeness. Areas outside these hypothesised regions were only considered significant if they survived correction at a threshold of *p* < 0.05 corrected.

## 3 Results

### 3.1 Behavioural

Immediately following the scanning session, participants were debriefed, and several aspects of their performance were probed. Debriefing ratings confirmed that in the construction condition, participants found it easy (1 = easy…5 = difficult) to start each trial with a “blank slate” in their imagination in readiness for constructing the scene (mean 1.16, *SD* 0.50). Once the narrative had began, participants found it easy (1 = easy…5 = difficult) to imagine a single acontextual first element (mean 1.89, *SD* 1.24), and easy to spatially integrate the subsequent elements into the scene (mean 1.63, *SD* 1.06). The constructed scenes were imagined from a first-person perspective (1 = 1st person…5 = 3rd person; mean 1.47, *SD* 1.07) and with the participant absent from the scene (1 = involved…5 = absent; mean 4.84, *SD* 0.50). In addition, scenes were rated with low emotional salience (1 = neutral…5 = emotional; mean 1.68, *SD* 0.95), moderate familiarity (1 = novel…5 = familiar; mean 2.89, *SD* 1.04), high plausibility (1 = low…5 = high; mean 4.18, *SD* 0.90) and with moderate coherence (1 = incoherent…5 = coherent; 3.26, *SD* 1.19). Participants rated maintaining all the elements within a scene as moderately difficult (1 = easy…5 = difficult; mean 3.58, *SD* 0.94). In line with the task instructions, participants kept to the narrative description without adding extra elements or contexts (1 = kept to narrative…5 = added new elements; 1.89, *SD* 1.04). In the control task, participants rated that the phrases elicited minimal imagery (1 = low…5 = high; mean 1.42, *SD* 0.61) and memory representations (1 = low…5 = high; mean 1.68, *SD* 0.69).

**Fig. 2:**
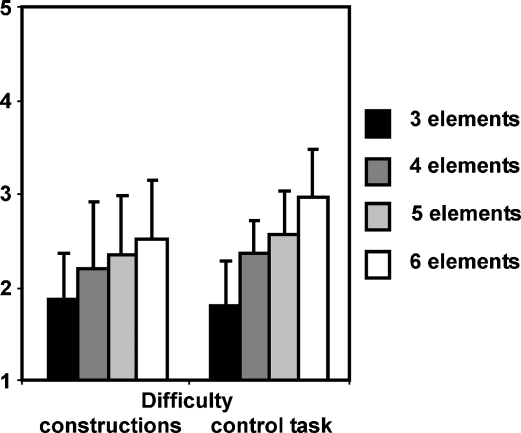
Mean difficulty ratings during scanning. As expected, difficulty (1 = easy…5 = difficult) increased with increasing numbers of elements in construction trials. This was mirrored by ratings for the control trials (see also Section 2 and Table S1 in Supplementary Materials).

During scanning, for the control task, participants were asked whether a particular word was present or absent in a trial. Performance was good, with an average of 92.03% (*SD* 7.85) correct for trials with 3 elements, 86.55% (13.11) for 4 element trials, 85.89% (10.51) for 5 element trials and 82.66% (11.37) for trials with 6 elements (combined over yes and no responses). For the construction conditions, participants rated three aspects of their performance at the end of each trial (see Table S1 in Supplementary Materials for full details of rating scores). Difficulty ratings were also taken at the end of each control trial. Overall, construction trials were rated as being vivid (mean 3.72, *SD* 0.60) and with coherently integrated elements (mean 3.4, *SD* 0.74). Difficulty ratings in construction trials and control trials were equivalent (construction: mean 2.24, *SD* 0.65; control: mean 2.42, *SD* 0.62–see Fig. 2). This was confirmed by a repeated-measures ANOVA testing factors of task (construction, control) and element number (3, 4, 5, 6) which showed that difficulty was well matched between construction and control trials [*F*(1,18) = 2.723, *p* = 0.116]. In both types of tasks, unsurprisingly, difficulty increased as the number of elements increased [*F*(3,54) = 41.991, *p* < 0.001] and this effect was greater for control trials compared to construction trials [*F*(3,54) = 5.960, *p* = 0.001]. Post hoc analyses revealed that difficulty significantly increased between adjacent elements 3 and 4 [*F*(1,18) = 34.252, *p* < 0.001]; 4 and 5 [*F*(1,18) = 4.520, *p* = 0.048]; and 5 and 6 [*F*(1,18) = 13.589, *p* = 0.002].

**Fig. 3:**
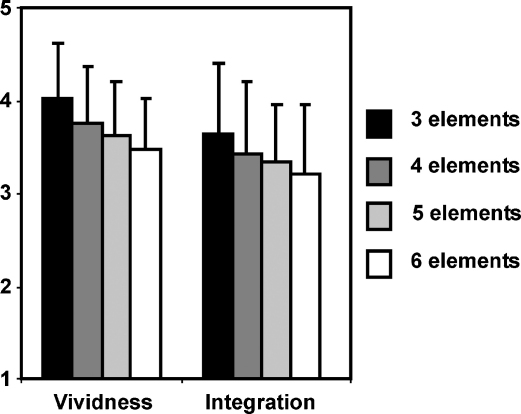
Mean ratings of vividness and perceived integration during scanning. Vividness (1 = not vivid…5 = very vivid) and perceived integration between the elements (1 = not integrated…5 = very integrated) decreased with increasing numbers of elements in construction trials (see also Sections 2 and 3 and Table S1 in Supplementary Materials).

In contrast to the ratings of difficulty, both vividness and the perceived integration between elements actually decreased as the number of elements in a scene increased (vividness: [*F*(3,54) = 11.075, *p* < 0.001]; integration: [*F*(3,54) = 4.190, *p* = 0.01] – see Fig. 3). Post hoc analyses showed that vividness ratings were significantly different between elements 3 and 4 [*F*(1,18) = 7.698, *p* = 0.013] but not between elements 4 and 5 [*F*(1,18) = 2.406, *p* = 0.138] or elements 5 and 6 [*F*(1,18) = 2.778, *p* = 0.113]. For perceived integration, the effect was driven by a difference between the more distant element numbers (3 and 6 [*F*(1,18) = 10.142, *p* = 0.005]; 4 and 6 [*F*(1,18) = 4.413, *p* = 0.05]; 3 and 5 [*F*(1,18) = 5.477, *p* = 0.031]), rather than between adjacent elements (3 and 4 [*F*(1,18) = 2.142, *p* = 0.161]; 4 and 5 [*F*(1,18) = 0.335, *p* = 0.570]; 5 and 6 [*F*(1,18) = 0.1626, *p* = 0.218]).

### 3.2 Neuroimaging

For all the contrasts reported here, each construction type always had its corresponding control trial type subtracted out first. This ensured that difficulty, attention, working memory load, and effort were controlled when making comparisons between construction conditions.

We first asked what happens in the context of scene construction when participants visualised the first element (1E-1Econtrol). This engaged areas that included LOC, dorsolateral prefrontal cortex, and ventrolateral prefrontal cortex. Interestingly, the hippocampus, parahippocampal gyrus and retrosplenial cortex were also significantly active during the visualisation of the first scene element (see Table 2). The addition of a second element [(2E-2Econtrol) > (1E-1Econtrol)], when participants were required to start integrating the elements together, resulted in increased activity in angular gyrus, IPS, and inferior and middle frontal gyri (see Table 3). Of note, there was no significant increase in activity in medial temporal or retrosplenial cortices. When a third element was added [(3E-3Econtrol) > (2E-2Econtrol)], however, the hippocampus, parahippocampal gyrus and retrosplenial cortex were once again significantly active, along with the LOC and frontal areas noted above (see Table 4).

Thus it would seem that activity in some regions such as IPS and angular gyrus increased with the addition of second and third elements. This was further confirmed by the contrast of element 3 with element 1 [(3E-3Econtrol) > (1E-1Econtrol)], which showed greater activation in these areas for element 3 than the first element (see Table 5). By contrast, areas such as the hippocampus, parahippocampal gyrus and retrosplenial cortex seemed to favour specific points in the scene construction process. They were significantly active for elements 1 and 3, but not for element 2, as evidenced in the lack of differences in these areas when elements 1 and 3 were compared (Table 5), and increased activity when either element 3 (Table 4) or element 1 (Table 6) was compared to element 2.

To ensure that these results were not simply due to differences in the control conditions, we investigated which regions were more active during the control tasks compared with the construction conditions [(1Econtrol-1E); (2Econtrol-2E); (3Econtrol–3E)]. All three contrasts showed similar patterns of activity, namely in dorsal occipital cortex [example peak: −36, −81, 9 (*z* = 3.64)], and left [−54, 0, −30 (*z* = 4.31)] and right [51,0, −27 (*z* = 3.84)] anterior temporal cortex. Of note, none of these regions overlapped with our regions of interest for scene construction. Moreover, none of our regions of interest were more active in 2Econtrol > 2E compared to the other two conditions, showing that differences between control trials do not explain the distinct patterns of activity we observed during scene construction.

Beyond the third element, there were no significant differences between the fourth, fifth or sixth elements, so we collapsed across them for further analyses. There were no brain areas more active for elements 4/5/6 when compared with element 3. Comparison with element 1 showed left middle frontal gyrus, left retrosplenial cortex, bilateral IPS, and left angular gyrus more active for elements 4/5/6 (see Table 7(A)). Posterior parietal areas were also more active in comparison with the second element (see Table 7(B)).

**Fig. 4:**
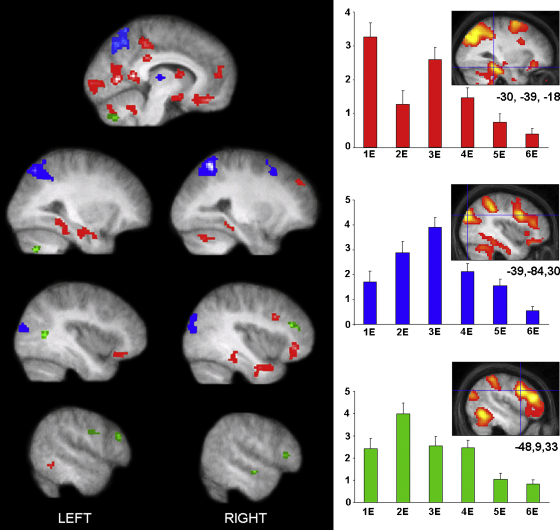
Time courses of activity in brain areas associated with scene construction. This figure summarises the main areas activated in the inter-element contrasts simultaneously on one set of brain slices, with the activations colour coded dependent on response profile. The activations (left panel) are thresholded here at *p* < 0.005 (uncorrected) for display purposes, and shown on the averaged structural MRI scan of the 19 participants. Areas in red, including left hippocampus, retrosplenial cortex, bilateral parahippocampal gyrus, had a biphasic response, being most active for elements 1 and 3. By contrast, areas shown in blue, including IPS and angular gyrus, had responses that peaked at element 3. While regions shown in green, including dorsolateral prefrontal and lateral temporal cortices, peaked at element 2. To the right are example beta plots for each response type taken from a contrast neutral with respect to element position (*all elements* > *all controls*), as well as inset images of the actual activations and exact coordinates from the neutral contrast. In the Supplementary Materials (Fig. S1) we include a range of other examples of beta plots derived from the *all elements* > *all controls* contrast to further illustrate the three response profiles. ‘E’ on the *x*-axis refers to the number of elements received at that point in the scene construction process. The *y*-axis represents arbitrary unit of the parameter estimates (betas). (For interpretation of the references to colour in this figure legend, the reader is referred to the web version of the article.)

Overall, the data are best summarised by looking at the time courses of the activations. Fig. 4 summarises the main areas activated in the inter-element contrasts simultaneously on one set of brain slices, with the activations colour coded dependent on response profile. We selected the coordinates of interest from the individual contrasts (as described in the tables above). In order to extract and plot the betas for each element, we sought the nearest activated voxel (closest local maxima; searching within a 3 mm radius around the peak coordinate) in a contrast that was neutral with regard to element position – we used the contrast of *all elements* > *all controls*. This ensured that the beta plot patterns were not biased by individual contrasts, but were also present within the overall scene construction network.

Three fMRI BOLD response profiles seemed to capture most of the data in this scene construction task. Some areas (red in Fig. 4) were activated by the first element, they were not significantly active for the second element, and then became involved once again for the third element. Areas with this biphasic profile included the anterior hippocampus, parahippocampal gyrus, retrosplenial cortex, LOC, medial prefrontal cortex, caudate nucleus, some parts of lateral temporal cortex, and areas within the cerebellum. By contrast, other regions had responses that rose up to a peak at the third element (blue in Fig. 4). These included the thalamus, superior frontal sulcus, IPS, medial parietal cortex, angular gyrus, another area within the cerebellum, and parts of occipital cortex. Finally, there were several areas that peaked at element 2, including lateral frontal regions, and a more lateral region of temporal cortex (green in Fig. 4).

#### 3.2.1 Additional analyses

We performed additional analyses to examine whether activity in any brain region was modulated by the behavioural rating scores of difficulty, vividness, and perceived integration between elements that were collected after each trial during scanning. Ratings of difficulty were not associated with significant increases in activity in any brain region. Increasing vividness was associated with activity in right parahippocampal gyrus (33, −21, −24; *z* = 4.15) and along the parieto-occipital sulcus (6, −60, 3; *z* = 3.41). Perceived integration between the elements was also associated with increases in similar areas (right parahippocampal gyrus: 30, −21, −27; *z* = 4.08; parieto-occipital sulcus: −9, −63, 12; *z* = 4.64; 9, −57, 3; *z* = 3.61), and also right ventromedial prefrontal cortex (6, 48, −18; *z* = 3.75).

## 4 Discussion

This study was designed to gain greater control over the construction or simulation of scenes, thought to be a key process underpinning functions such as episodic memory, thinking about the future, and navigation (Addis et al., 2007; Hassabis, Kumaran, & Maguire, 2007; Hassabis & Maguire, 2007; Hassabis & Maguire, 2009; Schacter & Addis, 2007; Spreng et al., 2009; Szpunar et al., 2007). We did this by devising a novel task where participants serially added and integrated elements together to form scenes of increasing complexity within their imagination. We found that three elements were sufficient to make a coherent and vivid scene, and once this was achieved, the addition of further elements seemed to involve only maintenance or small changes to that established scene. Both the object network and core brain network were active whilst participants constructed scenes. However the two networks did not operate separately, but rather, using this new task, we observed their interaction. In fact, scene construction was underpinned by three distinct sub-networks, encompassing regions within both object and core networks, each with different response profiles, favouring specific points in the scene construction process.

In previous studies, recall or imagination of single acontextual objects (i.e. in the absence of any background context) was associated with activation in LOC, IPS and DLPFC (Hassabis, Kumaran, & Maguire, 2007; Sugiura et al., 2005; Summerfield et al., 2009), areas distinct from regions activated by episodic memory and imagination of fictitious scenarios (Hassabis, Kumaran, & Maguire, 2007; Summerfield et al., 2009). In the present study, participants were instructed to start a trial with a ‘clean slate’ and imagine the first element as acontextual. Post-scan ratings confirmed that participants found it easy to do so. Imagination of this first element in the present study was indeed associated with activation of areas within the object network, e.g. LOC. However, areas within the core scene network were also active during imagination of the first element, including hippocampus, parahippocampal gyrus and retrosplenial cortex. Thus, the first element in the current task was effectively supported by a hybrid of the object and core scene networks. The main difference between this and previous studies that involved imagining single acontextual objects was the knowledge that the element was the starting point for a scene. Therefore, it seems that the mere intention to form a scene is enough to prime parts of the core network when only a single element is being imagined and, at least according to the participants, they are imagining that object without an explicit context.

Alternatively, it could be that the first element on a trial evoked a novelty response in areas such as the anterior hippocampus. However, this would also been the case when imagined objects were visualised in previous studies (e.g. Hassabis, Kumaran, & Maguire, 2007; Summerfield et al., 2009) and yet no hippocampal or other core network activations were apparent in those studies. It might be that the more widespread memory network activated for element 1 was associated with encoding, although the same could be argued for previous studies where no such activations were present (Hassabis, Kumaran, & Maguire, 2007; Summerfield et al., 2009). However, the present task embodied the known need to integrate the first element with perhaps as many as five subsequent elements. The activation of core network areas may reflect the active encoding of element 1 for later integration with future elements. The difficulty with this explanation concerns element 2.

If activity in areas such as the hippocampus reflected the encoding of element 1 for future integration, then it is likely that the same process would have been at play for element 2, because it too had the potential to be part of a scene with up to four additional elements. However, there was no significant increase in activity in the hippocampus or other core network areas during the imagination of the second element. This was the case when compared with element 1, but also, crucially, when compared with its baseline control task (see Table S2 in Supplementary Materials for details of the latter comparison). The lack of significant core network activity at element 2 is notable for two reasons. First, this finding could suggest that core network activation at element 1 was not simply due to encoding. If this is the case, then what might those activations reflect? It is interesting that those regions of the core network that were active at element 1, the hippocampus, parahippocampal gyrus and retrosplenial cortex, are known to be crucial for spatial processing (Bird & Burgess, 2008; Burgess, Maguire, & O’Keefe, 2002; Hassabis et al., 2009; Maguire, 2001). One speculation might be that the intention to construct a scene could be associated with the retrieval of possible scene templates or relate to some kind of scene-setting process (Epstein & Ward, 2010; Gronau, Neta, & Bar, 2008; O’Keefe & Nadel, 1978), although it is not clear how this fits with participants’ assertions that they imagined element 1 acontextually. Further work is clearly required to examine directly the effect of the intention to construct scenes, and its influence on retrieval of contextual associations (Bar, 2004; Bar & Aminoff, 2003; Fenske, Aminoff, Gronau, & Bar, 2006; Gronau et al., 2008).

The lack of significant core network activation, particularly in the hippocampus, during element 2 is somewhat surprising for a second reason. The construction task required participants to integrate elements together, to relate them in order to form a coherent scene. This task is clearly associative, and the hippocampus is widely held to be involved in making associations (Düzel et al., 2003; Goh et al., 2004; Mayes, Montaldi, & Migo, 2007; Peters, Daum, Gizewski, Forsting, & Suchan, 2009) and in relational processing (Cohen & Eichenbaum, 1993; Staresina & Davachi, 2009). It is clear from our data, however, that the hippocampus and a number of the other areas activated for element 1, were actually down-regulated with the addition of element 2. This suggests that the hippocampus (and other core network regions) are not invariably concerned with making associations, even in the context of a highly relational task. Instead, activation in these areas is not tonic throughout scene construction, showing that construction likely involves different processes that are engaged and disengaged as required. A similar response profile for the hippocampus was noted during navigation in a complex virtual reality city, where hippocampal activation was evident primarily in the first few seconds of route planning (Spiers & Maguire, 2006; see also Addis et al., 2007). In that study, lateral frontal areas were then recruited after the initial involvement of the hippocampus. In our study also, DLPFC increased activity with the introduction of element 2. This may reflect the holding of the first two elements in working memory (e.g. D’Esposito, Postle, & Rypma, 2000).

The phasic nature of the contribution of the hippocampus and other core network areas was further emphasised with the introduction of element 3. Many of the brain regions that had been active for element 1, but down-regulated for element 2, became significantly active once more. It is notable that not only did activity in areas that peaked at element 1 peak again at element 3, but activity in additional areas such as medial parietal cortex, IPS, angular gyrus and thalamus was also greatest at element 3. Moreover, with the addition of the third element, participants confirmed in the debriefing that ‘sceneness’ had been achieved, and this is where ratings of vividness and integration among scene elements were highest, and ratings correlated with activity in several of the regions activated, such as parahippocampal gyrus.

The question that naturally arises is whether the brain areas active at element 3 are performing the same functions as they were during element 1. The overall up-regulation of these areas, along with the feedback from participants that element 3 marked the achievement of sceneness, contrasts starkly with imagination of element 1, and suggests that for some brain regions at least, different functions may be in operation at different points during scene construction. We speculate that activation of, for instance, the hippocampus at element 3 may index the active construction and integration of the elements and the realisation of the concomitant spatial context (Hassabis, Kumaran, Vann, et al. 2007). It is interesting to contrast the biphasic response of brain areas like the hippocampus with regions where activity built up across elements to a peak at element 3. Activity in IPS, for example, may reflect the demands placed on visual attention (Coull & Frith, 1998; Lepsien & Nobre, 2006; Serences, Schwarzbach, Courtney, Golay, & Yantis, 2004; Yantis & Serences, 2003) and visuospatial working memory (Lepsien, Griffin, Devlin, & Nobre, 2005; Roth & Courtney, 2007; Roth, Serences, & Courtney, 2006) associated with holding online an increasingly complex scene. Clearly there is much scope for future studies to interrogate these finding further, not only using fMRI, but also by employing this new task to test patients with focal damage, such as those with bilateral hippocampal damage who are known to be impaired at imagining complex whole scenes (Hassabis, Kumaran, Vann, et al. 2007).

Perhaps surprisingly, beyond the third element, doubling the number of scene components resulted in increased activation in a relatively constrained set of primarily posterior parietal brain areas, presumably reflecting increased working memory and attentional load (Lepsien et al., 2005; Roth & Courtney, 2007; Roth et al., 2006). This finding also has parallels in the trial-by-trial vividness and integration scores which decreased with the addition of elements 4/5/6. Whilst overall the levels of vividness and integration were high, as noted above, ratings were highest by the third element, with vividness ratings significantly different between elements 3 and 4 but not between elements 4, 5 and 6. In the debriefing, participants confirmed that once element 3 had been included, the scene was ‘there’, that ‘sceneness’ had been achieved. Thereafter, participants felt like they were merely adding more elements to an already established scene, and somehow this felt less vivid than achieving sceneness after element 3.

In summary, this study was not designed to discern the specific functions of individual brain areas. Rather, our goal was to understand more about how the transition is made between an object (element) network (Hassabis, Kumaran, & Maguire, 2007; Sugiura et al., 2005; Summerfield et al., 2009) and a core (scene construction) network (Addis et al., 2007; Hassabis, Kumaran, & Maguire, 2007; Summerfield et al., 2009), and to break these networks down into smaller sub-networks and examine how they might be engaged at different points in the scene construction process. We succeeded in identifying three sub-networks with distinct profiles. It is possible that while individual brain areas within each sub-network perform different functions, they nevertheless operate as a functional unit during scene construction. We speculate that this may reflect priming of the core network at element 1 and perhaps scene-setting. This is followed by an interim period where an additional component is held in working memory, before scene construction proper and full sceneness is achieved with three elements. Thereafter, the scene is maintained without much additional activation in the networks. We believe the construction task developed here, providing more control to the experimenter, and less easily influenced by episodic memory, may be a useful paradigm for the future, as other kinds of elements could be included and manipulated. It will be important to explore the issues raised by our findings, and also consider how they might be linked with the networks that are emerging using other approaches such as resting state functional connectivity analyses (Vincent, Kahn, Snyder, Raichle, & Buckner, 2008; Wig, Buckner, & Schacter, 2009), in order to deconstruct a core brain network which is known to underpin crucial functions such as episodic memory, navigation and simulation of the future.

## Figures and Tables

**Table 1:** Example stimuli.

Construction phrases	
3 Elements	“*a dark blue carpet*”… “*a carved chest of drawers*”… “*an orange striped pencil*”
4 Elements	“*a wooden bench*”… “*a plain beige carpet*”… “*a small black cabinet*”… “*a pair of woolly gloves*”
5 Elements	“*a clear glass desk*”… “*a white electric fan*”… “*a polished marble floor*”… “*a canvass director's chair*”… “*a silver CD rack*”
6 Elements	“*a brown parquet floor*”… “*a metal waste paper basket*”… “*a maroon leather sofa*”… “*a beige patterned wall*”… “*a round low glass table*”… “*a small carriage clock*”

Control phrases	
3 Elements	“*a close description*”… “*a sum investigation*”… “*an altitude force*”
4 Elements	“*a complement similarity*”… “*an edge foundation*”… “*a secure approach*”… “*an inhibition clearance*”
5 Elements	“*a patience mineral*”… “*a notice route*”… “*an interest ambition*”… “*a content necessity*”… “*a compared moment*”
6 Elements	“*a series legality*”… “*a main variety*”… “*a style occupation*”… “*a sampler company*”…*.* “*a calculus purpose*”… “*a rarity growth*”

**Table 2:** Visualising the first element.^a^.

Region	Peak coordinate (*x*, *y*, *z*)			*Z*
Left middle frontal gyrus	−48	33	18	5.66
Right middle frontal gyrus	48	27	15	3.57
	42	9	27	5.01
Left ventrolateral prefrontal cortex	−42	24	−6	3.54
	−33	30	−18	4.59
Right ventrolateral prefrontal cortex	39	33	−18	5.13
Left preSMA	−3	18	54	3.71
Left superior frontal sulcus	−30	6	60	3.95
Left caudate nucleus	−18	−3	21	3.39
Left anterior hippocampus	−18	−12	−27	3.78
Left anterior hippocampus/perirhinal cortex	−21	0	−27	3.73
Right parahippocampal gyrus	39	−12	−33	4.88
	33	−36	−18	4.70
Left parahippocampal gyrus	−33	−36	−18	7.60
Left retrosplenial cortex	−9	−48	9	5.65
Left angular gyrus	−33	−81	36	4.81
	−42	−72	21	4.09
Right angular gyrus	39	−81	27	3.90
Left lateral occipital complex	−54	−51	−18	7.53
Right lateral occipital complex	51	−48	−24	3.63
Right occipital cortex	15	−102	−3	4.75
Left cerebellum	−45	−63	−33	3.33
Right cerebellum	30	−63	−33	6.91
	30	−69	−48	4.25
	9	−81	−39	3.78

a 1E > 1Econtrol.

**Table 3:** Adding a second element.^a^.

Region	Peak coordinate (*x*, *y*, *z*)			*Z*
Left middle frontal gyrus	−39	33	18	3.78
	−27	21	36	3.90
Right middle frontal gyrus	45	36	15	4.55
Left inferior frontal sulcus	−60	15	24	3.66
Right inferior frontal gyrus	57	24	9	3.50
Right superior frontal gyrus	18	15	45	3.74
Left inferior precentral sulcus	−42	3	36	4.21
Left putamen	−27	9	−6	4.57
Right superior temporal gyrus	63	−9	−9	3.86
Right superior temporal sulcus	36	−87	18	3.59
Left intra-parietal sulcus	−27	−63	42	4.60
Right precuneus	15	−63	51	3.90
Left angular gyrus	−27	−75	27	3.87
	−33	−69	18	4.38
Left occipital cortex	−30	−81	−3	3.72
	−36	−96	9	3.84
Left cerebellum	−18	−63	−42	4.29
	−9	−75	−36	3.68
Right cerebellum	3	−66	−6	3.66

a (2E-2Econtrol) > (1E-1Econtrol).

**Table 4:** Adding a third element.^a^.

Region	Peak coordinate (*x*, *y*, *z*)			*Z*
Left frontal pole	−24	57	0	3.46
Right middle frontal gyrus	18	57	30	3.41
	30	39	24	4.21
Right superior frontal sulcus	27	24	54	3.43
Right post-central gyrus	12	−57	69	3.84
Left ventrolateral prefrontal cortex	−30	30	−18	3.98
Right ventrolateral prefrontal cortex	60	12	−3	3.84
	39	39	−6	3.80
Right subgenual prefrontal cortex	9	24	−15	4.21
Left putamen	−27	9	9	5.15
Left middle temporal gyrus	−51	0	−27	3.46
Right middle temporal gyrus	54	−3	−27	4.49
Left anterior hippocampus	−24	−12	−24	3.70
Right anterior hippocampus/perirhinal cortex	27	0	−21	3.73
	27	−6	−36	3.71
Left parahippocampal gyrus	−21	−27	−18	4.07
	−33	−39	−15	3.67
Right parahippocampal gyrus	42	−36	−21	3.48
Right mid-cingulate cortex	21	−42	30	4.16
Left precuneus	−6	−63	51	4.34
	−6	−72	33	3.97
Right precuneus	15	−78	48	3.84
Left supramarginal gyrus	−57	−42	45	4.48
Right supramarginal gyrus	54	−36	42	4.08
Left retrosplenial cortex	−15	−45	−3	3.47
Right retrosplenial cortex	18	−48	12	4.78
Right lateral occipital complex	57	−48	−3	3.43
Right parieto-occipital sulcus	3	−66	15	3.73
Right cuneus	21	−78	21	4.10
Right lingual gyrus	21	−81	3	3.66
Left cerebellum	−45	−66	−33	3.95
	−30	−66	−45	3.35
Right cerebellum	18	−66	−42	3.62
	3	−72	−24	3.50

^a^(3E-3Econtrol) > (2E-2Econtrol).

**Table 5:** Comparing third and first elements.^a^.

Region	Peak coordinate (*x*, *y*, *z*)			*Z*
Right superior frontal sulcus	24	9	51	3.79
	30	3	60	3.36
Left thalamus	−15	−24	9	3.89
Left intra-parietal sulcus	−15	−66	54	5.67
	−9	−66	57	4.66
	−33	−45	45	3.93
Right intra-parietal sulcus	39	−54	57	3.53
	36	−39	39	3.76
Left angular gyrus	−42	−84	27	3.71
Right angular gyrus	39	−87	18	3.96
Left superior temporal sulcus	−36	−81	12	3.79

No areas were more active for: (1E-1Econtrol) > (3E-3Econtrol).

a (3E-3Econtrol) > (1E-1Econtrol).

**Table 6:** First element > second element.^a^.

Region	Peak coordinate (*x*, *y*, *z*)			*Z*
Right medial prefrontal cortex	3	51	18	4.30
Right middle frontal gyrus	24	42	45	3.62
Right inferior frontal sulcus	36	21	30	4.24
Left ventrolateral prefrontal cortex	−33	27	−21	3.82
Right ventrolateral prefrontal cortex	39	39	−9	4.44
	6	39	−9	4.38
Left preSMA	−6	−3	72	3.63
Left caudate nucleus	−18	−3	21	3.92
Left anterior hippocampus/perirhinal cortex	−21	0	−27	4.17
Left parahippocampal gyrus	−33	−39	−15	4.22
Left middle temporal gyrus	−51	0	−27	5.21
Right middle temporal gyrus	42	−3	−27	3.96
Right inferior temporal gyrus	48	−33	−18	3.24
Right temporal pole	39	15	−42	3.86
Right anterior mid-cingulate cortex	3	15	24	3.50
Post-central gyrus	0	−33	45	4.13
Right mid-cingulate cortex	15	−33	33	4.26
Left posterior cingulate cortex	−6	−54	33	3.83
Left retrosplenial cortex	−9	−48	9	5.13
Right retrosplenial cortex	18	−51	15	4.03
Left temporal parietal junction	−48	−57	33	3.38
Left lingual gyrus	−18	−72	3	3.56
Right occipital cortex	15	−102	−3	4.20
Right parieto-occipital sulcus	3	−69	15	5.18
Left cerebellum	−9	−51	−30	3.50
Cerebellum	0	−66	−30	3.58
Right cerebellum	30	−63	−33	3.85

a (1E-1Econtrol) > (2E-2Econtrol).

**Table 7:** (A) 4/5/6 elements > first element^a^ and (B) 4/5/6 elements > second element.^b^.

Region	Peak coordinate (*x*, *y*, *z*)			*Z*
(A)				
Left middle frontal gyrus	−36	39	12	3.88
Left retrosplenial cortex	−12	−39	15	4.01
Right intra-parietal sulcus	33	−39	36	3.83
	21	−72	54	5.21
Left intra-parietal sulcus	−15	−75	54	5.18
Left angular gyrus	−33	−69	18	4.15

(B)				
Left post-central gyrus	−6	−51	69	4.01
Right post-central gyrus	15	−57	69	4.00
Right intra-parietal sulcus	30	−54	51	3.64
Left precuneus	−6	−57	51	3.53
Left caudate nucleus	−18	−3	24	3.43

a [(4E-4Econ) + (5E-5Econ) + (6E-6Econ)] − (1E-1Econ).

b [(4E-4Econ) + (5E-5Econ) + (6E-6Econ)] − (2E-2Econ).

## References

[bib1] Addis D.R., Wong A.T., Schacter D.L. (2007). Remembering the past and imagining the future: Common and distinct neural substrates during event construction and elaboration. Neuropsychologia.

[bib2] Bar M. (2004). Visual objects in context. Nature Reviews Neuroscience.

[bib3] Bar M., Aminoff E. (2003). Cortical analysis of visual context. Neuron.

[bib4] Bartlett F.C. (1932). Remembering.

[bib5] Bird C.M., Burgess N. (2008). The hippocampus and memory: Insights from spatial processing. Nature Reviews Neuroscience.

[bib6] Botzung A., Denkova E., Manning L. (2008). Experiencing past and future personal events: Functional neuroimaging evidence on the neural bases of mental time travel. Brain and Cognition.

[bib7] Buckner R.L., Andrews-Hanna J.R., Schacter D.L. (2008). The brain's default network: Anatomy, function, and relevance to disease. Annals of the New York Academy of Sciences.

[bib8] Buckner R.L., Carroll D.C. (2007). Self-projection and the brain. Trends in Cognitive Sciences.

[bib9] Burgess N., Maguire E.A., O’Keefe J. (2002). The human hippocampus and spatial and episodic memory. Neuron.

[bib10] Cohen N.J., Eichenbaum H. (1993). Memory, amnesia and the hippocampal system.

[bib11] Conway M.A., Pleydell-Pearce C.W. (2000). The construction of autobiographical memories in the self-memory system. Psychological Review.

[bib12] Coull J.T., Frith C.D. (1998). Differential activation of right superior parietal cortex and intraparietal sulcus by spatial and nonspatial attention. Neuroimage.

[bib13] D’Esposito M., Postle B.R., Rypma B. (2000). Prefrontal cortical contributions to working memory: Evidence from event-related fMRI studies. Experimental Brain Research.

[bib14] Düzel E., Habib R., Rotte M., Guderian S., Tulving E., Heinze H.J. (2003). Human hippocampal and parahippocampal activity during visual associative recognition memory for spatial and nonspatial stimulus configurations. Journal of Neuroscience.

[bib15] Epstein R.A., Ward E.J. (2010). How reliable are visual context effects in the parahippocampal place area?. Cerebral Cortex.

[bib16] Fenske M.J., Aminoff E., Gronau N., Bar M. (2006). Top-down facilitation of visual object recognition: Object-based and context-based contributions. Progress in Brain Research.

[bib17] Goh J.O., Siong S.C., Park D., Gutchess A., Hebrank A., Chee M.W. (2004). Cortical areas involved in object, background, and object-background processing revealed with functional magnetic resonance adaptation. Journal of Neuroscience.

[bib18] Gronau N., Neta M., Bar M. (2008). Integrated contextual representation for objects’ identities and their locations. Journal of Cognitive Neuroscience.

[bib19] Hassabis D., Chu C., Rees G., Weiskopf N., Molyneux P.D., Maguire E.A. (2009). Decoding neuronal ensembles in the human hippocampus. Current Biology.

[bib20] Hassabis D., Kumaran D., Maguire E.A. (2007). Using imagination to understand the neural basis of episodic memory. Journal of Neuroscience.

[bib21] Hassabis D., Kumaran D., Vann S.D., Maguire E.A. (2007). Patients with hippocampal amnesia cannot imagine new experiences. Proceedings of the National Academy of Sciences USA.

[bib22] Hassabis D., Maguire E.A. (2007). Deconstructing episodic memory with construction. Trends in Cognitive Science.

[bib23] Hassabis D., Maguire E.A. (2009). The construction system of the brain. Philosophical Transaction of the Royal Society London B Series: Biological Science.

[bib24] Lepsien J., Griffin I.C., Devlin J.T., Nobre A.C. (2005). Directing spatial attention in mental representations: Interactions between attentional orienting and working-memory load. Neuroimage.

[bib25] Lepsien J., Nobre A.C. (2006). Cognitive control of attention in the human brain: Insights from orienting attention to mental representations. Brain Research.

[bib26] Maguire E.A. (2001). The retrosplenial contribution to human navigation: A review of lesion and neuroimaging findings. Scandinavian Journal of Psychology.

[bib27] Mayes A., Montaldi D., Migo E. (2007). Associative memory and the medial temporal lobes. Trends in Cognitive Sciences.

[bib28] O’Keefe J., Nadel L. (1978). The hippocampus as a cognitive map.

[bib29] Okuda J., Fujii T., Ohtake H., Tsukiura T., Tanji K., Suzuki K. (2003). Thinking of the future and past: The roles of the frontal pole and the medial temporal lobes. Neuroimage.

[bib30] Peters J., Daum I., Gizewski E., Forsting M., Suchan B. (2009). Associations evoked during memory encoding recruit the context-network. Hippocampus.

[bib31] Raichle M.E., MacLeod A.M., Snyder A.Z., Powers W.J., Gusnard D.A., Shulman G.L. (2001). A default mode of brain function. Proceedings of the National Academy of Sciences USA.

[bib32] Roth J.K., Courtney S.M. (2007). Neural system for updating object working memory from different sources: Sensory stimuli or long-term memory. Neuroimage.

[bib33] Roth J.K., Serences J.T., Courtney S.M. (2006). Neural system for controlling the contents of object working memory in humans. Cerebral Cortex.

[bib34] Rubin D.C., Schrauf R.W., Greenberg D.L. (2003). Belief and recollection of autobiographical memories. Memory and Cognition.

[bib35] Schacter D.L., Addis D.R. (2007). The cognitive neuroscience of constructive memory: Remembering the past and imagining the future. Philosophical Transaction of the Royal Society London B Series: Biological Science.

[bib36] Schacter D.L., Addis D.R., Buckner R.L. (2008). Episodic simulation of future events: Concepts, data, and applications. Annals of the New York Academy of Sciences.

[bib37] Schacter D.L., Norman K.A., Koutstaal W. (1998). The cognitive neuroscience of constructive memory. Annual Review of Psychology.

[bib38] Serences J.T., Schwarzbach J., Courtney S.M., Golay X., Yantis S. (2004). Control of object-based attention in human cortex. Cerebral Cortex.

[bib39] Spiers H.J., Maguire E.A. (2006). Thoughts, behavior, and brain dynamics during navigation in the real world. Neuroimage.

[bib40] Spreng R.N., Mar R.A., Kim A.S. (2009). The common neural basis of autobiographical memory, prospection, navigation, theory of mind, and the default mode: A quantitative meta-analysis. Journal of Cognitive Neuroscience.

[bib41] Staresina B.P., Davachi L. (2009). Mind the gap: Binding experiences across space and time in the human hippocampus. Neuron.

[bib42] Sugiura M., Shah N.J., Zilles K., Fink G.R. (2005). Cortical representations of personally familiar objects and places: Functional organization of the human posterior cingulate cortex. Journal of Cognitive Neuroscience.

[bib43] Summerfield J.J., Hassabis D., Maguire E.A. (2009). Cortical midline involvement in autobiographical memory. Neuroimage.

[bib44] Szpunar K.K., Watson J.M., McDermott K.B. (2007). Neural substrates of envisioning the future. Proceedings of the National Academy of Sciences USA.

[bib45] Vincent J.L., Kahn I., Snyder A.Z., Raichle M.E., Buckner R.L. (2008). Evidence for a frontoparietal control system revealed by intrinsic functional connectivity. Journal of Neurophysiology.

[bib46] Wig G.S., Buckner R.L., Schacter D.L. (2009). Repetition priming influences distinct brain systems: Evidence from task-evoked data and resting-state correlations. Journal of Neurophysiology.

[bib47] Yantis S., Serences J.T. (2003). Cortical mechanisms of space-based and object-based attentional control. Current Opinion in Neurobiology.

