# Supplementary material for: Differential engagement of brain regions within a ‘core’ network during scene construction
Source: Neuropsychologia. 2010 Apr;48(5):1501–9. doi: 10.1016/j.neuropsychologia.2010.01.022 (PMC2850391; doi:10.1016/j.neuropsychologia.2010.01.022)
Supplement: Supplementary file 1 [file mmc1.doc]

**Differential engagement of brain regions within a ‘core’ network during scene construction**

Jennifer J. Summerfield, Demis Hassabis, Eleanor A. Maguire

**Supplementary Materials**

| **Table S1: Mean behavioural rating scores during fMRI** | | | | |
| --- | --- | --- | --- | --- |
|  | **Vividness** | **Integration** | **Difficulty (Construction)** | **Difficulty (Control)** |
| **3 elements** | 4.03 (0.59) | 3.64 (0.77) | 1.87 (0.50) | 1.80 (0.48) |
| **4 elements** | 3.75 (0.62) | 3.41 (0.79) | 2.21 (0.69) | 2.37 (0.35) |
| **5 elements** | 3.63 (0.57) | 3.34 (0.62) | 2.34 (0.63) | 2.55 (0.46) |
| **6 elements** | 3.48 (0.54) | 3.21 (0.75) | 2.53 (0.61) | 2.97 (0.51) |

1 = low…5 = high; SD in brackets

| **Table S2: Imagining two elements compared with the control task*** | | | | | | |
| --- | --- | --- | --- | --- | --- | --- |
| Region |  | Peak coordinate (x, y, z) | | | | Z |
|  |  |  |  |  |  |  |
| Left middle frontal gyrus |  | -45 | 9 | 33 |  | <8.00 |
| Right middle frontal gyrus |  | 45 | 33 | 15 |  | 7.38 |
| Right ventrolateral prefrontal cortex |  | 24 | 27 | -18 |  | 3.96 |
| PreSMA |  | 0 | 12 | 51 |  | 4.86 |
| Left superior frontal suclus |  | -24 | -3 | 54 |  | 5.68 |
| Right superior frontal sulcus |  | 24 | 6 | 54 |  | 3.47 |
| Left putamen |  | -27 | 9 | -9 |  | 4.25 |
| Left intraparietal sulcus |  | -15 | -75 | 51 |  | 7.40 |
| Right intraparietal sulcus |  | 33 | -48 | 51 |  | 3.39 |
|  |  | 30 | -72 | 42 |  | 5.02 |
| Left lateral occipital complex |  | -51 | -63 | -9 |  | 7.15 |
| Right lateral occipital complex |  | 60 | -57 | -18 |  | 4.07 |
| Left cerebellum |  | -9 | -78 | -36 |  | 4.54 |
| Right cerebellum |  | 15 | -84 | -39 |  | 4.24 |
|  |  | 36 | -72 | -42 |  | 4.21 |
|  |  |  |  |  |  |  |
| *2E > 2Econtrol |  |  |  |  |  |  |


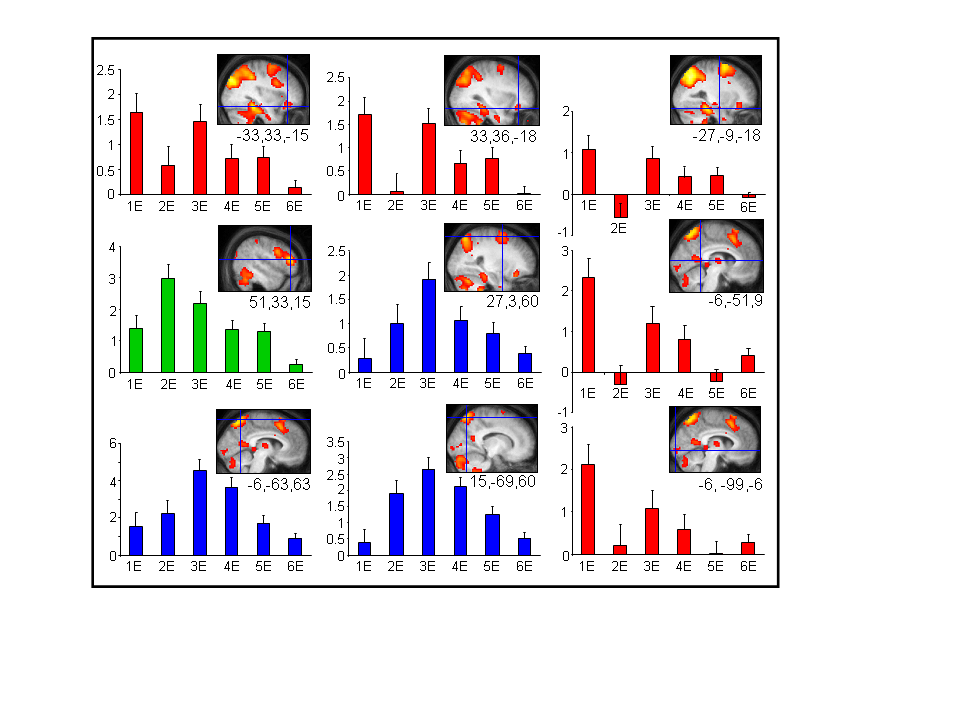


**Fig. S1. Further examples of beta plots for regions of interest from the schematic in Figure 4.**

As outlined in Figure 4, three patterns of activation were apparent in the data (shown in red, blue, and green). Figure 4 included one example beta plot for each response type. Here we show additional beta plots taken from a contrast neutral with respect to element position (*all elements > all controls*), as well as inset images of the actual activations and exact coordinates from the neutral contrast. ‘E’ on the x-axis refers to the number of elements received at that point in the scene construction process. The y-axis represents arbitrary unit of the parameter estimates (betas).
